# Effectiveness of additional trunk exercises on gait performance: study protocol for a randomized controlled trial

**DOI:** 10.1186/s13063-017-1989-1

**Published:** 2017-06-02

**Authors:** Tamaya Van Criekinge, Wim Saeys, Ann Hallemans, Luc Vereeck, Willem De Hertogh, Patricia Van de Walle, Nathalie Vaes, Christophe Lafosse, Steven Truijen

**Affiliations:** 1 Department of Rehabilitation Sciences and Physiotherapy, Faculty of Medicine and Health Sciences, University of Antwerp, Universiteitsplein 1, 2610 Antwerp, Belgium; 2 KU Leuven Department of Psychology, University of Leuven, Leuven, Belgium; 3 Scientific Unit RevArte, Rehabilitation Hospital RevArte, Antwerp, Belgium; 4 Department of Experimental Psychology, Ghent University, Ghent, Belgium

**Keywords:** Trunk, Stroke, Balance, Gait, Rehabilitation, Core stability

## Abstract

**Background:**

Evidence is lacking concerning the effect of additional trunk rehabilitation on gait performance. Investigating gait performance by both clinical and biomechanical outcome measures might lead to new scientific insights into the importance of the trunk during gait rehabilitation in people suffering from stroke. This protocol was written according to the SPIRIT 2013 Statement.

**Methods and design:**

An assessor-blinded randomized controlled trial will be conducted in patients with impaired trunk control after stroke. A total of 60 patients will be randomly allocated to the control or the experimental group by means of sealed opaque envelopes. They will receive either 16 h of additional trunk exercises (experimental group) or cognitive exercises (controls) for 1 h a day, 4 days a week for 4 weeks. Patients will also receive 2 h of standard care consisting of physiotherapy and occupational therapy. Gait performance will be assessed clinically by the Tinetti Test and biomechanically by means of a full body gait analysis. In addition, the effect of the exercise protocol on the trunk itself and trunk activities of daily living will be assessed by the Trunk Impairment Scale and the Barthel Index.

**Discussion:**

Despite the evidence demonstrating the importance of trunk control after stroke, studies about the effects of trunk rehabilitation on gait performance are inconsistent. In the current study, a more sophisticated treatment protocol will be used to enlarge therapeutic improvements, the relationship between clinical and biomechanical measures of gait performance can be investigated, and the sustainability of the effects of trunk exercises over time will be examined. Since clinical improvements are of greater importance to patients and physiotherapists, clinical assessment scales will be used as primary outcome measures.

**Trial registration:**

ClinicalTrials.gov, ID: NCT02708888. Registered on 2 March 2016.

**Electronic supplementary material:**

The online version of this article (doi:10.1186/s13063-017-1989-1) contains supplementary material, which is available to authorized users.

## Background

Several studies have demonstrated that truncal function is impaired in patients suffering from stroke. Therefore, it is of utmost importance to no longer neglect truncal function during rehabilitation. Impairments in truncal function are characterized by a diminished sitting balance, decreased trunk coordination, reduced trunk control and lower trunk muscle strength, and altered trunk position sense [1–5]. Subsequently, patients displayed increased lateral movements, but decreased vertical movements compared to healthy controls [6]. Rehabilitation programs, such as core stability training, sitting and reaching training, which aim to reduce these impairments, seemed to improve clinical measures of static and dynamic sitting and standing balance after stroke [7–10].

However, more research is necessary regarding the effect of trunk rehabilitation on gait performance. Cabanas-Valdes et al. (2013) concluded that although trunk training exercises could be an effective rehabilitation strategy for improving trunk performance, further confirmation is necessary with respect to gait performance [11]. Since gait performance can be measured by means of clinical and biomechanical assessment it is of interest to incorporate both assessment methods. This is to make sure that every aspect of walking is assessed. Although biomechanical analysis gives a more in-depth understanding of the biomechanical improvements in gait performance, clinical improvements are of greater importance to patients and physiotherapists as they assess functional tasks and are easier to use in a clinical setting.

The trunk has long been defined as a so-called passenger unit since it was suggested that it is carried by the lower limbs instead of actively contributing to ambulation [12]. Yet, a more recent study suggested that the role of the passenger unit in pathological gait must be recognized in the decrease of gait efficiency in neurological patients [13]. The trunk is, in fact, one of the main contributors to the increased mechanical work of the passenger unit. However, as the trunk is one of the main contributors to decreased gait, it should no longer be defined as a passenger unit. Therefore, the effects of additional trunk exercises on the trunk itself during ambulation should be examined more thoroughly.

Consequently, this study aims to explore the effects of additional trunk exercises on gait performance, as measured clinically and biomechanically, in people suffering from stroke who have been submitted to a rehabilitation hospital.

### Objectives

The primary objective of this study is to examine the effect of an additional trunk exercise program on gait performance after stroke. Primarily, clinical assessment of gait performance is made by the Tinetti Test (TT) and its subscales; secondarily, biomechanical assessment by a full body gait analysis. Trunk performance will be assessed by means of the Trunk Impairment Scale (TIS) and the independency of a patient’s performance concerning activities of daily living (ADL) by the Barthel Index (BI). Patients suffering from stroke will be receiving either 16 h of additional trunk exercises or the same duration of cognitive exercises for a period of 4 weeks.

The second objective is to explore the sustainability of the effects of trunk exercises over time. It is important to know whether the treatment effects are sustainable over time or if continuous therapeutic input is necessary to maintain the level of functioning even after patients are discharged home.

## Methods and design

### Study design and setting

The design of this study is a 4-week, assessor-blinded randomized controlled trial with a 1-month follow-up at an established rehabilitation hospital. Participants will be randomly allocated to either the experimental or the control group by simple randomization executed by an independent researcher who is not involved in the assessment or treatment of the patients. After participants agree to participate in this study, the independent researcher will draw one of the 60 envelopes (30 for each group) and will assign the participants to the allocated group. Patients will be recruited from the stroke population of the rehabilitation hospital RevArte, located in Edegem (Antwerp, Belgium). This rehabilitation hospital is a 194-bed facility and is able to offer inpatient and outpatient rehabilitation for approximately 300 patients at the same time. All participants will receive 16 h of either trunk or cognitive exercises in addition to the multidisciplinary standard care stroke rehabilitation program provided by the rehabilitation staff.

### Participants

#### Inclusion criteria

Patients will have to meet the following eligibility criteria to be included in the study: (1) a hemorrhagic or ischemic stroke diagnosis, confirmed on the basis of computed tomography (CT) imaging or magnetic resonance imaging (MRI), (2) no known history of previous stroke, (3) stroke onset within the previous 5 months, and (4) patients are between 18 and 85 years of age.

#### Exclusion criteria

Patients will be excluded from the study if: (1) they scored 20 or higher on the TIS which indicates normal truncal function [14]. Since patients should be able to improve their trunk performance by means of trunk rehabilitation, normal trunk performance should be excluded [14], (2) they scored lower than 2 on the Functional Ambulation Categories (FAC) as patients need to be able to ambulate without physical support to ensure that gait analysis can be executed safely, (3) they are not able to sit independently, without support and supervision, for 30 s on a stable surface. Patients will have to perform trunk exercises on unstable surfaces; therefore, a minimum truncal function is necessary to ensure a safe environment during training, (4) they suffer from other neurological and orthopedic disorders that could influence motor performance and balance, (5) they are unable to understand verbal instructions. The cognitive and communicative abilities of the patients will be assessed by a neuropsychologist or speech therapist of the rehabilitation hospital RevArte, and (6) they are patients over the age of 85 years because an hour of intense therapy will be too demanding for this population. Additionally, unpublished data from our research group suggest that involvement of the trunk during walking clearly changes after the age of 80 years. This decision was made to exclude gait patterns that resemble a geriatric gait pattern instead of a hemiplegic pattern. Although the patient population needed for this study is specific to patients who are able to walk and have an impaired truncal function, based on unpublished data, it is anticipated that approximately 35% of all hospitalized stroke patients will meet these criteria.

### Procedures

#### Recruitment and selection

**Fig. 1:**
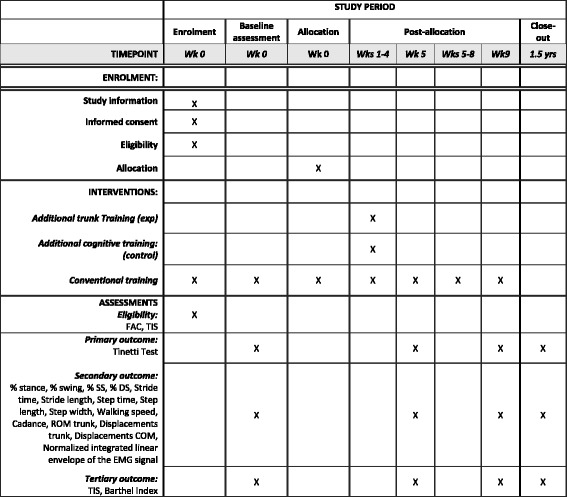
The schedule of enrollment, interventions, and assessments. Abbreviations: *wk* week, *wks* weeks, *yrs* years, *exp* experimental, *FAC* Functional Ambulation Categories, *TIS* Trunk Impairment Scale, *SS* single support, *DS* double support, *ROM* range of motion, *COM* center of mass

The time schedule of enrollment, assessment, interventions, and follow-up according to the Standard Protocol Items: Recommendations for Interventional Trials (SPIRIT) guidelines can be found in Fig. 1. Patients are recruited and screened for eligibility in three consecutive steps. Firstly, the treating physiotherapists will be thoroughly briefed concerning the inclusion and exclusion criteria of the study since they will be providing the researchers with the information for possible inclusion. Next, study information will be given to potential participants by the main researcher. This comprises the objective and description of the study, the duration of the study, and its risks and benefits. If the patients are interested in the study, an appointment will be made to provide more detailed information and to answer possible questions. When the patient agrees to participate in the study, the informed consent will be signed before obtaining medical record admission to guarantee privacy. Lastly, after obtaining informed consent the patients will be screened by the primary investigator to assure inclusion by means of the TIS and the FAC. Information concerning stroke diagnosis, medical history, and stroke onset will be acquired from patient records.

#### Baseline assessment

Clinical information comprises date of birth, type of stroke, location of stroke, medical history, drug therapy, and the use of orthosis and assistive devices. Both biomechanical and clinical assessment will be performed prior to intervention. The clinical assessment will consist of the following clinical tests: the Tinetti Test (TT); the Trunk Impairment Scale (TIS), Functional Ambulation Categories (FAC), and the Barthel Index (BI). The procedures of the clinical tests can be found in the studies of Tinetti et al. (1986), Verheyden et al. (2004), Holden et al. (1984), and Mahoney et al. (1965) [15–18]. The biomechanical assessment procedure will consist of a 3D, full body gait analysis which will be executed at the M^2^OCEAN movement analysis laboratory (Multidisciplinary Motor Centre Antwerp, University of Antwerp, Antwerp, Edegem). A VICON analysis system (VICON© Motion Systems Ltd., London, UK) with a measuring frequency of 100 Hz and a measurement error smaller than 1 mm and 1° will be used to measure kinematic parameters in all three planes; sagittal, frontal, and transversal. Eight infrared automated cameras (VICON T10 cameras, 100 fps, 1 Megapixel) will measure the 3D coordinates of the reflective markers. In addition, initial contact and toe off will be defined based on the ankle trajectories of the reflective markers together with 3 AMTI type OR 6-7 force plates (1000 fps, 46 × 50 × 8 cm) and one AccuGait® (1000 fps) force plate recording. The movement analysis laboratory is equipped with a 16-channel telemetric wireless surface electromyography (EMG) system (1080 Hz; Cometa, Rome, Italy) which measures muscle activity of the trunk and lower limb muscles. Recordings will be analyzed using the VICON Nexus 1.8.5. software and the Plug-In-Gait software package as clinical model. The joint rotation angles will be calculated from the YXZ Cardan angles derived by comparing the relative orientations of the two segments. For example, the knee angles will be calculated from the femur and the untorsioned tibial segments relative to the fixed laboratory axis. Subsequently, data will be reconstructed and the reflective markers will have to be labeled. After labeling, the data will be filtered to eliminate any recorded noise by means of a low-pass Butterworth filter (second order, zero phase, cutoff frequency 6 Hz). Furthermore, gait cycle events and parameters will be calculated from the filtered ankle marker trajectories. Data will be saved as c3d files and further processed in MATLAB® (The MathWorks, Inc., Natick, MA, USA) by means of customized MATLAB® scripts.

Gait analysis will commence by preparing the patient in a standardized manner. Firstly, the following anthropometrics will be determined to make an adequate 3D model of the patient: body height, body weight, leg length, shoulder width, thickness of the wrist and of the second metacarpophalangeal joint, distance between the medial and lateral humeral epicondyles, between the medial and lateral condyles of the femur, and between the medial and lateral malleoli. In addition, clinical assessment will be performed to assess the underlying problem of the abnormal gait pattern. A correctly executed clinical assessment is necessary to interpret the information provided by the full body gait analysis. The following characteristics will be assessed: (1) passive ROM of the hip, knee, ankle, and foot will be assessed by means of goniometry, (2) inspection of the foot (e.g., varus, valgus, claw toes, mid-foot break), (2) muscle length of the rectus femoris and hamstrings by means of the Duncan-Ely Test and the popliteal angle [19, 20], (3) muscle strength by means of manual muscle testing according to the guidelines of the Oxford Medical Research Council [21]. Isometric muscle force will be assessed during open-chain movements by providing manual resistance against these movements. The patient’s effort is graded on a scale of 0 to 5 of which 0 represents no observed movement and 5 represents normal muscle contraction against maximal resistance, (4) selectivity of movements is the ability to perform isolated joint movements without using flexor or extensor patterns or undesired movements at other joints. Selectivity of movements will be either normal, impaired, or unable, (5) muscle tone will be assessed by means of the Modified Tardieu Scale [22, 23]. This test quantifies spasticity by assessing the resistance to applied stretch at specified velocities. A score of 0 to 5 can be given implying no increase of resistance to rigid movement of the limb, respectively. In addition, the presence of clonus will be assessed, (5) sensitivity will be assessed by the revised Nottingham Sensory Assessment Scale [24]. Tactile sensation (light touch, pressure, and pin prick) and sharp-dull discrimination will be assessed at defined points of contact. Proprioception will be assessed to see if the patient is able to detect movement and in which direction, and (6) the Confusion Test, which assesses the selectivity of hip flexion movement and the force of the dorsal flexor muscles in a flexion synergy, will be performed [25].

Secondly, disposable gel electrodes (Kendall^TM^, 30 mm × 24 mm) will be applied after anthropometric measurements. Before application of the electrodes the skin will be properly prepared, by shaving and degreasing, to ensure a good electrode-skin contact and to minimize the risk of artefacts. Electrodes will be placed on the left and right musculus rectus femoris, musculus vastus lateralis, musculus biceps femoris, musculus semitendinosus, musculus tibialis anterior, musculus gastrocnemius and musculus erector spinae during maximal muscular contraction according to the SENIAM guidelines [26].

**Fig. 2:**
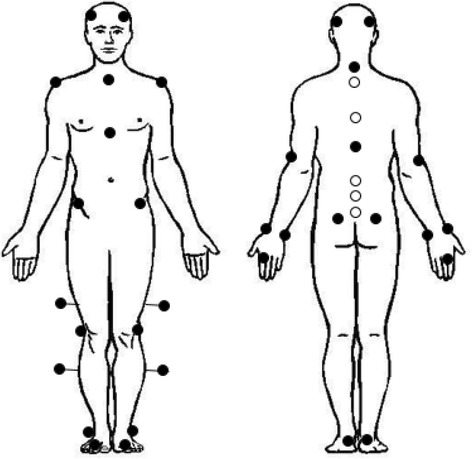
Plug-In-Gait model (*black*) and spine model (*white*)

Thirdly, reflective markers will be placed on bony anatomical landmarks according to the standard Plug-In-Gait model combined with a more detailed spine model developed and assessed for reliability by Heyrman et al. (2013) [27]. These two models allow the computation of linear and angular displacements of the different body segments. Although, good intra-protocol repeatability has been established for the Plug-In-Gait model, variability due to differences in marker placement has shown to be the major contributor to overall variance [28, 29]. Therefore, a standardized protocol was implemented whereby bony landmarks were located by manual palpitation by the primary investigator. To diminish artefacts, the motion trackers were firmly affixed to the skin with double-sided tape. Reflective markers for the Plug-In-Gait model of the upper body are located at the front and back of the head (left and right), the processus spinosus (PS) of the seventh cervical vertebrae, the PS of the tenth thoracic vertebrae, the jugular notch of the sternum, the xiphoid process of the sternum, the left and right acromioclavicular joints, the left and right points of rotation of the elbow, the styloid process of the left and right ulna and radius, and on the dorsum of both hands just below the second metacarpals. Reflective markers for the Plug-In-Gait model of the lower body are located on the left and right spina iliaca anterior superior and spina iliaca posterior superior, the left and right points of knee rotation, the left and right lateral malleoli, the second left and right metatarsal heads, and the left and right calacanei. Reflective markers for the spine model are located on the PS of the second and sixth thoracic vertebrae, and the PS of the first, third, and fifth lumbar vertebrae. Four markers are attached at one third of the length of the femur and fibula in alignment with trochanter major, the rotation point of the knee, and the lateral malleolus to examine the presence of rotation in the lower limbs (Fig. 2).

Finally, EMG sensors will be attached to the surface electrodes. The signal-to-noise ratio will be checked to ensure clear EMG recordings.

When preparation is complete, a static calibration will be performed with the knee alignment device (KAD) to avoid knee joint angle cross-talk. Patients are asked to stand still in the middle of the force platform with arms outstretched and thumbs facing down. The KAD will be placed at the rotation axis of the knee. To ensure minimal cross-talk the KAD will be replaced three times by two different investigators. During analysis the most optimal placement of the KAD will be chosen to analyze the dynamic walking trials. Subsequently, the patients will have to walk barefoot at their natural, self-selected speed and without walking aid or orthosis, if possible, over a 12-m walkway and supervised by a skilled physiotherapist in order to avoid falls or other problems. To ensure the patient’s safety during walking, a safety harness can be worn which will not limit trunk movement. In total, a minimum of six walking trials will be recorded. Of these six trials, three will consist of clean heel strikes onto the force platforms from the right stride and three from the left so that kinetic parameters can be analyzed.

#### Allocation

A blinded investigator will allocate patients to the control or the experimental group by means of concealed envelopes which will be kept off site.

#### Interventions

All patients will receive a multidisciplinary standard care stroke rehabilitation program provided by the rehabilitation staff of RevArte. The standard care program will be patient-specific and consists of 1 h of physiotherapy and 1 h of occupational therapy. Standard care will mainly consist of gait rehabilitation, muscle strengthening and activities to enhance motor control of the arms, legs, and trunk by applying appropriate motor relearning strategies. Additionally, all patients will be receiving 30 min of speech therapy and neuropsychological treatment when necessary. Thirty participants will be assigned to the exercise group focusing on trunk coordination, selectivity, and strengthening. In total, 30 patients will be distributed to the control group. The amount of additional therapy is based on results from a meta-analysis, where at least 16 h of augmented therapy is needed within the first 6 months after stroke to achieve a favorable effect on the activities of daily living (ADL) [30]. Patients will receive the additional therapy over a period of 4 weeks. Kwakkel et al. (2004) hypothesized that a high dose of task-specific exercises should be applied over a short period of time. Therefore, both groups will receive an additional therapy hour a day, 4 days a week over a short period of 4 weeks. Although both groups receive the same amount of therapeutic input, the specificity of the training programs will be different.

The control group will be receiving the same amount of therapeutic input relevant for subjects with stroke but without the specific focus on the trunk. However, the trunk and lower limbs are involved in a variety of motor activities such as reaching, sitting without support, and active mobilization exercises [31, 32]. Therefore, a useful task has to be performed by the control group without incorporating the trunk or lower limbs. Since approximately 30% of patients will be cognitively impaired in the 5 years following a stroke, patients will be challenged to perform cognitive activities for the same amount of time [33]. For this, the RevArte Visual Search Test (RVST) of Lafosse et al. (2013) and the Visuospatial Neglect Test Battery (VNTB) of Vaes et al. (2015) will be executed by the patients. A thorough description of these tests can be found in Lafosse et al. (2013) and Vaes et al. (2015) [34, 35]. The tests are run on the Metrisquare DiagnoseIS software platform and are presented on a Wacom pen display with a large active screen area of 47.70 × 26.82 cm (total screen size of 56.39 × 37.34 cm), connected to a PC. The dual screen technology enables the researcher to observe and designate interim results at their own computer screen while the participant uses the pen at the display surface. During each of the 4 weeks, patients will perform both the VNTB and RVST, consisting of two 1-h sessions for each test battery. To prevent truncal activity the cognitive exercises will not incorporate activities where a large mechanical perturbation is induced as a positive correlation was shown between the amplitude of arm movement and anticipatory postural adjustments of the trunk [36]. Before and after the additional cognitive exercises, patients will describe their global fatigue on a Visual Analogue Fatigue Scale with 0 being fully awake and 10 being extremely tired.

The additional training for the experimental group will focus on increasing trunk control. The exercise program will consist of task-specific movements of the upper and lower part of the trunk both in the supine and sitting positions. However, we are well aware of the fact that several exercises focusing on trunk control also incorporate muscle activity of the lower limbs [31]. It is impossible to incorporate an efficient trunk exercise program with no involvement of the lower limbs. This will be achieved by executing exercises which will specifically focus on recruiting abdominal and back muscles during functional activities, strengthening of these muscles, and integrating the use of these core muscles into basic daily tasks [37]. Karthikbabu et al. (2011) concluded that trunk exercises performed on physio balls are more effective than those performed on plinths in improving trunk control and functional balance [38]. Therefore, we will be implementing a similar exercise program on unstable surfaces where patients will be receiving exercises for 25 min in a supine position followed by a 5-min resting period, and then a 30-min training session in a seated position (Table 1). Progression will be implemented in a standardized manner and determined by the physiotherapist based on the patient’s performance. The trunk exercises will always commence in the supine position, the physiotherapists need to take the safety of the patient into account when progressing to the sitting position. If safety is not guaranteed, exercises in supine will be repeated. Trunk exercises will be initiated with moderate assistance of the physiotherapist and gradually reduced to no assistance. As soon as possible, patients will have to execute the exercises with no contact between the feet and the ground to ensure a minimum of involvement of the lower limbs. Furthermore, intensity can be increased by implementing the following changes: (1) reducing base of support, (2) increasing the lever arm, (3) increasing limits of stability, (4) increasing the hold time, (5) increasing the number of repetitions, and (6) presence of visual feedback by executing the exercises with the eyes open or the eyes closed. In addition, patients will describe their global fatigue on a Visual Analogue Fatigue Scale, with 0 being fully awake and 10 being extremely tired, before and after the additional trunk exercise program.

**Table 1:** Trunk exercise program

Position	Exercises on stable surface	Exercise on unstable surface
Supine (25 min)	Selective flexion/extension of the lower trunk	Selective flexion/extension of the lower trunk
	Pelvic bridging: lifting pelvis in crook lying with both feet supported	Pelvic bridging: lifting pelvis with lower limbs supported on physio ball
	Unilateral pelvic bridging: lifting pelvis in crook lying with one foot supported	Unilateral pelvic bridging: lifting pelvis with one leg supported on physio ball
	Pelvic bridging with displacements: lifting pelvis in crook lying and placing pelvis left and right of midline	Pelvic bridging with displacements: lifting pelvis with lower limbs supported on physio ball and place pelvis left and right from midline
	Lower trunk rotation: moving the lower limbs from left to right in crook lying	Lower trunk rotation: moving the lower limbs from left to right with legs supported on physio ball
	Lower trunk flexion: lifting lower limbs symmetrically to chest in crook lying	Lower trunk flexion: moving the lower limbs symmetrically to chest with lower limbs supported on physio ball
	Upper trunk flexion: lifting shoulder girdle symmetrically in crook lying	Upper trunk flexion: lifting shoulder girdle symmetrically with lower limbs supported on physio ball
	Upper trunk flexion rotation: lifting shoulder girdle asymmetrically in crook lying	Upper trunk flexion rotation: lifting shoulder girdle asymmetrically with lower limbs supported on physio ball
	Lower trunk flexion rotation: lifting lower limbs asymmetrically to chest in crook lying	Lower trunk flexion rotation: moving the lower limbs asymmetrically to chest with lower limbs supported on physio ball
Sitting(30 min)	Selective flexion/extension of the lower trunk	Selective flexion/extension of the lower trunk while seated on physio ball
	Selective lengthening and shortening of one side of the trunk	Selective lengthening and shortening of one side of the trunk while seated on physio ball
	Upper trunk lateral flexion: initiating movement from the shoulder girdle	External and internal perturbations while seated on physio ball
	Lower trunk lateral flexion: initiating movement from the pelvic girdle	Upper trunk lateral flexion: initiating movement from the shoulder girdle while seated on physio ball
	Upper trunk rotation: moving each shoulder forward and backwards	Lower trunk lateral flexion: initiating movement from the pelvic girdle while seated on physio ball
	Forward reach: reaching the arms out forwards from the trunk	Upper trunk rotation: moving each shoulder forward and backwards while seated on physio ball
	Lateral reach: 'reaching the arms out sideways from the trunk	Weight shifting while seated on physio ball
	Shuffling forward and backward on hard surface	Forward reach: reaching the arms out forwards from the trunk while seated on physio ball
		Lateral reach: reaching the arms out sideways from the trunk while seated on physio ball

#### Post-treatment assessment and follow-up

Immediately after the intervention and 1 month post intervention, patients will again be subjected to a clinical and biomechanical assessment to examine treatment effect and the treatment effect sustainability.

#### Discontinuations

Patients who withdraw from the study prior to intervention and after giving consent will be reported as “did not receive intervention.” If they withdraw during the 4-week intervention phase, an intention-to-threat analysis will be performed. Analysis will be performed as if the subject received the treatment (or control condition). Reasons for withdrawal will be noted.

#### Blinding

Although we will try to blind patients, therapists, and assessors it is unlikely that patients and therapists will stay blind during the course of this study due to the nature of the applied treatment. However, to make sure that the risk of bias stays low, patients will be registered in the database by means of a patient ID code so assessors are blinded during analysis. Only the primary investigator will have knowledge regarding allocation.

### Outcome measures

#### Primary outcome measures

Gait performance as measured clinically via the TT is the primary outcome measure. The FAC will not be used as an outcome measure to assess gait performance as it is a more descriptive clinical measure and less detailed compared to the TT. The TT measures gait and balance using a 2- or 3-point ordinal scale with scores ranging from 0 to 1 or 2. The maximum score of the total TT is 28 points, whereby a maximum of 12 and 16 points can be obtained for gait and balance subscales, respectively. Nine items, such as sit to stand, standing balance with eyes open and eyes closed, turning 360° and sitting down, are assessed on the balance subscale. The gait subscales assess eight items ranging from step length, step symmetry, foot clearance, and step width. Reliability and validity of the TT for stroke patients have been reported (ICC2,1 = 0.84; TT-FIMmotor *r* = 0.55; TT-gait speed *r* = 0.82) [39]. The minimal clinically important difference of the TT is 7 points (area under the curve (AUC) = 0.743) and a minimal detectable change of 6 points [39].

#### Secondary outcome measures

Several outcome measures will be assessed during biomechanical gait analysis. Firstly, the following spatiotemporal parameters will be assessed: percentage (%) of stance time, % of swing time, % of single support time (SS), % of double support time (DS), stride time, step time, stride length, step length, step width, walking speed, and cadence. Secondly, kinematic parameters, which describe the displacements and/or range of motion (ROM) of the segments in the sagittal, frontal, and transversal planes, will be assessed. Kinematic parameters across the entire gait cycle will be examined by means of 1D Statistical Parametric Mapping (spm1d). Subsequently, horizontal and vertical displacements of the center of mass (COM) will be analyzed during right and left strides. Lastly, the normalized integrated linear envelope of the EMG signal will be computed, making it possible to observe both muscle timing and amplitude simultaneously. Raw EMG data will be rectified to obtain absolute values of the signal. Thereafter, the linear envelop, integrated EMG and normalized EMG, will be acquired by computing the outline of the signal, the AUC and calculating the average EMG signal throughout the gait cycle for every subject, respectively. EMG activity across the entire gait cycle will be examined by means of spm1d statistics. Muscle activity of the back, abdominal, and lower limb muscles will be registered. Two trials with a sufficient number of strides from each condition will be selected for analysis.

#### Tertiary outcome measures

Trunk Impairment Scale (TIS) and Barthel Index (BI), assessing trunk control and ADL, will be used as tertiary outcome measures. The TIS consists of three subscales assessing both static and dynamic sitting balance as well as trunk coordination. TIS scores range from a minimum of 0 to a maximum of 23; subscales score up to 7, 10, and 6 points, respectively. A higher score indicates better truncal function. The static sitting balance subscale assesses whether a person can sit independently and remain seated with their legs crossed. The dynamic sitting balance subscale assesses the ability to actively shorten each side of the trunk, initiated from either the shoulder girdle or the pelvic girdle. The trunk coordination subscale assesses the ability to rotate the shoulder girdle and the pelvic girdle [15]. Reliability, validity, and internal consistency of the TIS for stroke patients have been reported (ICC = 0.96; TIS-TCT *ρ* = 0.84; Cronbach’s *α* = 0.89) [1, 15, 40]. The BI is an index assessing the independency of a patient’s performance concerning the ADL. The maximum score of the BI gives a score out of 100 with increments of 5 points to assess whether the patient is fully dependent, independent, or needs some help regarding ten topics: feeding, bathing, grooming, dressing, toilet use, bowel and bladder continence, transferring, mobility, and stair climbing [41]. Reliability, validity, and internal consistency of the BI have been reported (ICC = 0.94; BI-FIMmotor *r* ≥ 0.92; *α* = 0.89–0.90) [42, 43]. Table 2 summarizes all the outcome measures described herein.

**Table 2:** Outline of the outcome measures

Outcome measure	Domain	Tool	Baseline	Post	Follow-up
Eligibility assessment					
FAC	Gait	Clinical	X		
Primary outcome measure					
Tinetti Test	Gait	Clinical	X	X	X
Secondary outcome measures					
% stance, % swing	Gait	Biomechanical, spatiotemporal	X	X	X
% SS, % DS	Gait	Biomechanical, spatiotemporal	X	X	X
Stride time	Gait	Biomechanical, spatiotemporal	X	X	X
Stride length	Gait	Biomechanical, spatiotemporal	X	X	X
Step time	Gait	Biomechanical, spatiotemporal	X	X	X
Step length	Gait	Biomechanical, spatiotemporal	X	X	X
Step width	Gait	Biomechanical, spatiotemporal	X	X	X
Walking speed	Gait	Biomechanical, spatiotemporal	X	X	X
Cadence	Gait	Biomechanical, spatiotemporal	X	X	X
ROM trunk	Gait	Biomechanical, kinematics	X	X	X
Displacements trunk	Gait	Biomechanical, kinematics	X	X	X
Displacements COM	Gait	Biomechanical, kinematics	X	X	X
Normalized integrated linear envelope of the EMG signal	Gait	Biomechanical, EMG	X	X	X
Tertiary outcome measures					
TIS	Trunk	Clinical	X	X	X
Barthel Index	ADL	Clinical	X	X	X

*FAC* Functional Ambulation Categories, *SS* single support, *DS* double support, *ROM* range of motion, *COM* center of mass, *EMG* electromyography, *TIS* Trunk Impairment Scale, *ADL* activities of daily living

### Sample size

Analysis was based on results of a previous randomized controlled trial concerning the effect of additional trunk exercises on the TT and TIS that was carried out by our research group [7]. Saeys et al. (2012) [7] reported a change score of the TT of 13.45 and 5.2 points for the experimental and control groups, respectively. The number of patients required for this study was calculated a priori to ensure sufficient statistical power. Analysis showed that a sample size of 30 patients in each group, 60 in total, was necessary to detect a difference with 80% using a two-tailed hypothesis (with significance level of *p* = 0.05).

### Statistical analysis

Descriptive data analysis will be performed for the collected variables of the participants. The Kolmogorov-Smirnov Test and visual inspection of the data will be performed to evaluate whether the data are normally distributed. Differences between the experimental and control groups for the clinical data will be evaluated by means of repeated measures (ANOVA). Level of significance will be set at *p* < 0.05. When the stroke population is too heterogeneous, stratified sampling will be done to divide the study population into more homogenous groups. Differences along the entire kinematic, kinetics, and EMG curves will be assessed by means of spm1d in MATLAB®. Statistical parametric mapping will make interference about the topological features of statistical processes that are continuous functions [44].

### Data collection and management

Standardized forms have been drafted to ensure accurate and reliable data collection regarding clinical information and assessment. Furthermore, multiple training sessions will be provided to the assessors and therapists to ensure standardized treatment, assessment, and data analysis. The amount of training will be dependent on the familiarization with the clinical scales and therapy techniques of the assessors and therapists. Standardized procedures have to be followed during assessment and treatment. Several meetings will be held where the primary investigator will be informed about current affairs and can be consulted if questions arise or problems occur. Collected data and information will be processed with the utmost discretion and anonymity; patient data will be registered by means of an identification number and not by name. The primary investigator will keep records of the patients’ clinical records and data, so patients can be contacted for follow-up assessment if they are already discharged. Patient records and data will be kept for 10 years after publication as suggested by the Ethics Review Committee of the University Hospital of Antwerp (UZA, Edegem, Antwerp, Belgium).

### Ethical considerations and dissemination

#### Ethics review

Ethical approval of the study has been obtained from the Ethics Review Committee of the University Hospital of Antwerp (UZA, Edegem, Antwerp, Belgium) and the Ethics Review Committee of the GasthuisZusters Hospital (GZA, Wilrijk, Antwerp, Belgium). The following reference numbers were used during the application: 15/42/433 and 151203ACADEM. The trial is registered in the electronic database for clinical trials (ClinicalTrials.gov; 2 March 2016; ID: NCT02708888).

#### Safety

Although we foresee no major risks or adverse effects, patients who are harmed during the course of this study will receive no compensation since treatment and assessment are applied as a health care service provided under national health insurance. If harm is caused by therapists or assessors, the appropriate insurance will cover the expenses.

#### Dissemination

The results of this study will be presented at several research conferences, published in peer-review journals, and will be included in the doctoral thesis of the primary investigator. Findings will be presented at several workshops and training days for practicing physiotherapists hosted by the University of Antwerp and located in several rehabilitation hospitals such as RevArte, care and nursing facilities.

## Discussion

The aim of this study is to further explore the effects of additional customized trunk exercises on clinical and biomechanical gait performance. Despite the evidence demonstrating the importance of trunk control after stroke, studies about the effects of trunk rehabilitation on gait performance are inconsistent. The findings of this study might lead to new scientific insights into the importance of the trunk during gait rehabilitation in people suffering from stroke submitted to a rehabilitation hospital. Since clinical improvements are of greater importance to patients and physiotherapists as they assess functional tasks and are easier to use in a clinical setting, the TT and its subscales will be the primary outcome measures. Subsequently, a variety of biomechanical parameters collected by a full body gait analysis will be our secondary outcome measures. However, it is still important to consider the effect of this training program on the trunk itself and on ADL as this is the main focus of the training program. Because of this, the TIS and BI will assessed as tertiary outcome measures.

Our trial has several strengths. Firstly, since both clinical and biomechanical outcome measures will be examined, it is of interest to take a closer look at the relationship between both assessment methods. Several biomechanical parameters, such as step symmetry, step width, and step length, are assessed by the TT. Investigating whether the clinically observed parameters are significantly different from the biomechanical parameters assessed in a gait laboratory might reveal whether these tests are sufficient to tell us something about gait performance. In addition, the TIS evaluates trunk control in a seated position. With the results of this study, it is possible to examine whether the TIS is able to predict trunk motion during walking. Secondly, a more sophisticated treatment protocol based on new scientific insights and a previous study within our research group will be used to enlarge therapeutic improvements [7]. The following important changes will be implemented in this training protocol: (1) a more intensive exercise program as it is hypothesized that a high dose of task-specific exercises should be applied over a short period of time. Therefore, both groups will receive additional therapy for 1 h a day, 4 days a week over a short period of 4 weeks, (2) this is an exercise program that is executed on unstable surfaces since Karthikbabu et al. (2011) reported short-term effects in favor of exercises on physio balls compared to plinth training [45]. Thirdly, the investigation of the sustainability of the effects of trunk exercises over time. It is important to know whether the treatment effects are sustainable over time or if continuous therapeutic input is necessary to maintain the level of functioning even after patients are discharged home. Lastly, the effect of trunk rehabilitation on biomechanical parameters has not yet been thoroughly examined.

However, there are a few limitations to consider. Blinding of therapists and patients will be unfeasible as the experimental and control therapies differ considerably. Subsequently, to our knowledge no research has been conducted to examine the effect of cognitive exercises on measures of balance and gait. Although, we assume that the control therapy will not reveal carry-over effects on balance an gait, we cannot say this without doubt. However, the exercises are drafted in a way that no carry-over will be expected. In addition, trunk exercises do not solely activate trunk muscles. Lower limb muscles will also be activated during seated reaching exercises [31]. It is almost impossible to eliminate lower limb activity during motor activities. Yet, the specificity of the training program concerns trunk control and activation of the upper and lower trunk.

After completion of this study we will have gained insights into the effects of trunk rehabilitation on clinical and biomechanical parameters of gait performance. This protocol was written according to the SPIRIT 2013 Statement [45]. The SPIRIT Checklist can be found as an additional file (see Additional file 1).

### Current study status

At the time of submission ethics approval has been granted. The study started recruiting patients in 2016. Recruitment of the study is still ongoing and, so far, 28 patients have been recruited for this trial. We anticipate that 18 months (2017) will be needed to complete the trial.

## Additional file

Additional file 1: SPIRIT Checklist. (DOC 120 kb)

## Abbreviations

ADL: Activities of daily living
AUC: Area under the curve
BI: Barthel Index
COM: Center of mass
DS: Double support
EMG: Electromyography
FAC: Functional Ambulation Categories
KAD: Knee alignment device
PS: Processus spinosus
ROM: Range of motion
RVST: Revarte Visual Search Test
Spm1d: One-dimensional Statistical Parametric Mapping
SS: Single support
TIS: Trunk Impairment Scale
TT: Tinetti Test
VNTB: Visuospatial Neglect Test Battery
